# Characterization of AMBN I and II Isoforms and Study of Their Ca^2+^-Binding Properties

**DOI:** 10.3390/ijms21239293

**Published:** 2020-12-05

**Authors:** Veronika Vetyskova, Monika Zouharova, Lucie Bednarova, Ondřej Vaněk, Petra Sázelová, Václav Kašička, Jiri Vymetal, Jaroslav Srp, Michaela Rumlová, Tatsiana Charnavets, Klara Postulkova, Janne E. Reseland, Kristyna Bousova, Jiri Vondrasek

**Affiliations:** 1 Institute of Organic Chemistry and Biochemistry of the Czech Academy of Sciences, Flemingovo nam. 2, 160 00 Prague, Czech Republic; veronika.vetyskova@uochb.cas.cz (V.V.); monika.vargova@uochb.cas.cz (M.Z.); lucie.bednarova@uochb.cas.cz (L.B.); petra.sazelova@uochb.cas.cz (P.S.); vaclav.kasicka@uochb.cas.cz (V.K.); jiri.vymetal@uochb.cas.cz (J.V.); jaroslav.srp@uochb.cas.cz (J.S.); klara.postulkova@uochb.cas.cz (K.P.); 2 Department of Biochemistry and Microbiology, University of Chemistry and Technology Prague, Technicka 5, 166 28 Prague, Czech Republic; 3 Second Faculty of Medicine, Charles University, V Uvalu 84, 150 06 Prague 5, Czech Republic; 4 Department of Biochemistry, Faculty of Science, Charles University, Hlavova 2030/8, 128 40 Prague, Czech Republic; ondrej.vanek@natur.cuni.cz; 5 Department of Biotechnology, University of Chemistry and Technology Prague, Technicka 5, 166 28 Prague, Czech Republic; michaela.rumlova@vscht.cz; 6 Institute of Biotechnology of the Czech Academy of Sciences, Prumyslova 595, 252 50 Vestec, Czech Republic; chernt@ibt.cas.cz; 7 Department of Biomaterials, Institute of Clinical Dentistry, University of Oslo, 0317 Oslo, Norway; j.e.reseland@odont.uio.no

**Keywords:** ameloblastin, biomineralization, oligomerization, calcium binding, intrinsically disordered protein (IDPs)

## Abstract

Ameloblastin (Ambn) as an intrinsically disordered protein (IDP) stands for an important role in the formation of enamel—the hardest biomineralized tissue commonly formed in vertebrates. The human ameloblastin (AMBN) is expressed in two isoforms: full-length isoform I (AMBN ISO I) and isoform II (AMBN ISO II), which is about 15 amino acid residues shorter than AMBN ISO I. The significant feature of AMBN—its oligomerization ability—is enabled due to a specific sequence encoded by exon 5 present at the N-terminal part in both known isoforms. In this study, we characterized AMBN ISO I and AMBN ISO II by biochemical and biophysical methods to determine their common features and differences. We confirmed that both AMBN ISO I and AMBN ISO II form oligomers in in vitro conditions. Due to an important role of AMBN in biomineralization, we further addressed the calcium (Ca^2+^)-binding properties of AMBN ISO I and ISO II. The binding properties of AMBN to Ca^2+^ may explain the role of AMBN in biomineralization and more generally in Ca^2+^ homeostasis processes.

## 1. Introduction

Ameloblastin (Ambn; also known as amelin or sheatlin) is an enamel matrix phosphoprotein classified by computational and biophysical methods as an intrinsically disordered protein (IDP) [1,2,3]. Ambn with other ameloblasts-specific IDPs is important in biomineralization processes during enamel formation [4,5,6]. Interestingly, tooth enamel in vertebrates is the hardest known mineralized tissue [7]. The biomineralization is a complex process controlled by ameloblasts, which are the specific secretory cells responsible for the synthesis of enamel matrix proteins that control the process leading to the formation of enamel matrix [8]. In addition to Ambn, there are other Extracellular Matrix Proteins (EMPs) classified as IDPs involved in the mineralization process—amelogenin [9], tuftelin [10], and enamelin [11]. There are complementary proteases acting during mineralization; the most important are kallikrein-4 (KLK4) and enamelysin (MMP20) [12]. It is known that IDPs do not form a stable three-dimensional structure, neither elements of secondary structures (with some exception upon a dramatic change of environment [13]) nor their biological function is primarily dependent on interactions with other biomolecules [4]. However, some of the IDPs can adopt different structures upon a binding to their native partner. In such case, we describe these complexes as “fuzzy”, as they can provide more options of binding between the interacting partners [4,14].

The sequence-based analysis of human ameloblastin (AMBN) according its chemical–physical properties implies an existence of two different parts of the protein. The N-terminal part (AMBN-Nterm)—about 220 amino acids residues long (spanning regions from exon 1 to exon 10) is neutral, enriched by proline-rich regions and a few short regions of hydrophobic amino acids. This suggests that these hydrophobic regions can act in AMBN-facilitated protein–protein interactions. The AMBN C-terminus (AMBN-Cterm) is highly charged, 225 amino acids residues long (spanning the regions from exon 10 to exon 13), and its total calculated charge is −19.6 [1,15,16,17,18]. This finding clearly sets a distinction between both parts of the AMBN. Additionally, the AMBN-Cterm highly enriched by negatively charged amino acid residues may also act as a cation-binding proxy [6,16]. Mineralized tooth enamel is an inorganic structure composed of hydroxyapatite (HAP) crystals, which are formed by a saturated solution of HAP (Ca_10_(PO_4_)_6_(OH)_2_) [4,19,20]. This raises the question of whether the negatively charged part of AMBN—the acidic AMBN-Cterm (unphosphorylated or phosphorylated)—may play a role in calcium (Ca^2+^) binding and to what extent it may contribute to the oligomerization of AMBN and the biomineralization process.

The first supramolecular assemblies of enamel have been shown to be formed by specific protein–protein interactions of proteins secreted by ameloblasts during the enamel formation process [4,21,22] with a pivotal role of Ambn and Amel. It was proposed that this arrangement could be important for scaffolding suitable for the deposition of hydroxyapatite [22]. It was also shown experimentally that Ambn and Amel can co-assemble in a specific order facilitated by a tyrosyl-binding motif [22,23,24], promoting the formation of hetero-oligomers [2,4,21,22]. Interestingly, the AMBN itself exists only in oligomeric states, and their population is heterogeneous [2]. It was proved that the AMBN-specific oligomerization property is maintained by exon 5, which is a 37 amino acid long region at the AMBN-Nterm [1,2,25]. Some residues of exon 5 that are evolutionarily conserved facilitate interaction with AMEL and also binding to a lipid membrane [24,25,26]. AMBN with deleted exon 5 (AMBN del E5) completely loses its ability to form oligomers and exists only in a monomeric form [2]. The exon 5 knock-out has a significant biological effect. The produced monomeric AMBN in the mice model causes structural abnormalities that result in an irregular formation of dental enamel [25]. Defects in the process of enamel development result in amelogenesis imperfecta [27].

Extracellular matrix proteins (EMPs) originated from a common ancestor and form an evolutionarily conserved family of Secretory Calcium-Binding Phosphoproteins (SCPPs). Most scientists today believe that they have enamel-only functions and are produced exclusively by secretory ameloblast cells [28]. However, the evolutionary divergence of EMPs [28,29] indicates a discrepancy between the evolutionary appearance of the enamel and the divergence of the EMP. A possible explanation for this contrast is, as suggested by [30], that EMPs may initially be co-evolved for parallel functions, e.g., roles in general Ca^2+^ metabolism and/or its deposition in bone tissues explosion when Ca^2+^ storage has become an important evolutionary innovation [31].

Both Ambn ISO I and Ambn ISO II isoforms are evolutionary conserved and have been identified at the mRNA or cDNA level in human and three other mammalian species (Rattus rattus, Mus musculus, Sus scrofa domestica) [15,32,33,34]. The human AMBN is known to be expressed in most mammalian cells in two isoforms: AMBN ISO I and AMBN ISO II. The AMBN ISO II is 15 amino acids shorter than AMBN ISO I, and interestingly, these residues are the first amino acids of exon 6 at its very N-terminus [1,15,32]. So, there is a question if due to the important role of exon 5, the missing piece of the sequence of exon 6 could influence the oligomerization properties of the AMBN. Moreover, predicted phosphorylation sites at tyrosine or serine residues in this segment (YEYSLPVHPPPLPSQ) suggest important implications for both isoforms and their functions [3,15,33]. There is no information about the importance or different functions of both AMBN isoforms nor their different physical–chemical properties. In this study, we addressed properties of human AMBN ISO I and AMBN ISO II using a spectrum of biophysical methods, and the assessed ability of both isoforms to bind Ca^2+^ as one of the consequences of protein oligomerization.

## 2. Results

### 2.1. AMBN ISO I and AMBN ISO II Belong to IDPs

**Figure 1:**
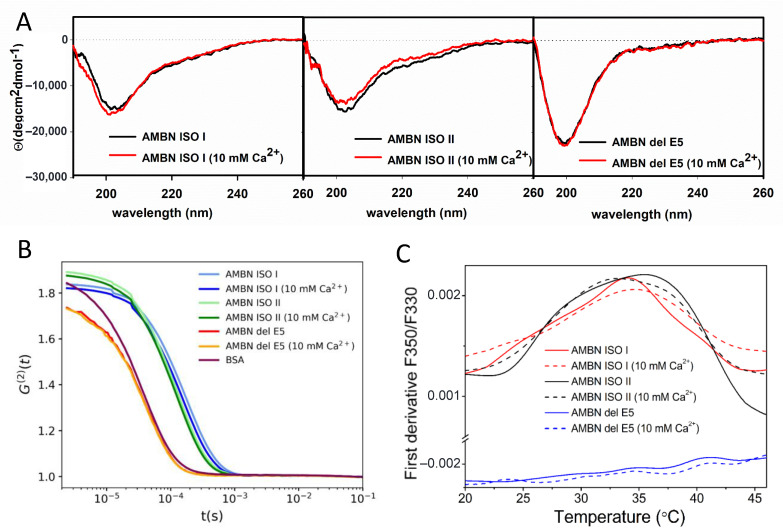
(**A**) Circular dichroism (CD) spectra of ameloblastin full-length isoform I (AMBN ISO I), ameloblastin full-length isoform II (AMBN ISO II) and AMBN with deleted exon 5 (AMBN del E5). Sample before (black) and after (red) addition of 10 mM CaCl_2_. (**B**) Analysis of autocorrelation functions (ACF) from dynamic light scattering (DLS) spectra of AMBN ISO I, AMBN ISO II, AMBN del E5, and bovine serum albumin (BSA) as a standard for globular protein. ACF of proteins in the absence or presence of 10mM CaCl_2_. (**C**) First derivative of thermal unfolding curves of AMBN ISO I, AMBN ISO II, and AMBN del E5 in the presence or absence of 10 mM CaCl_2_.

The circular dichroism (CD) spectra of AMBN ISO I (Figure 1A and Figure S1) were consistent with our previous AMBN characterizations [2]. The CD spectrum of AMBN ISO I was characterized by a negative maximum at ≈202 nm (Figure S1) accompanied by negative shoulder at ≈220 nm. With the temperature increase, the 202 nm spectral band is red shifted, and its intensity decreases. This spectral change is accompanied by an intensity increase of the negative spectral band at ≈220 nm. The observed spectral changes with an isosbestic point at ≈208 nm are typical for IDPs [35]. The overall low spectral intensity of CD spectra was in good agreement with our previous analysis [2], which was interpreted as a raising presence of β-structure elements.

The CD spectrum of AMBN ISO II has similar spectral shape as observed for AMBN ISO I, with a negative maximum at 203 nm and negative shoulder at ≈220 nm (Figure 1A and Figure S1). This spectral band is red shifted upon the increasing temperature accompanied by an increase of negative spectral band intensity at ≈220 nm similarly as AMBN ISO I. The corresponding isosbestic point was determined at ≈209 nm.

The CD spectrum of AMBN del E5 is characterized by a negative maximum at ≈199 nm with a higher intensity than AMBN ISO I and AMBN ISO II (Figure 1A and Figure S1). The overall spectral changes together with the isosbestic point at ≈208 nm support our conclusion that AMBN del 5 is an IDP [35].

The CD spectra of AMBN ISO I and AMBN ISO II in 10mM Ca^2+^ solution are in fact almost unchanged compared with the Ca^2+^-free solution. The spectral changes for AMBN ISO I and ISO II upon increasing temperature in the 10 mM Ca^2+^ solution do not provide clear localization of the isosbestic point. Interestingly, both proteins are dominantly populated by β-structure elements at 90 °C, suggesting a high aggregation state. With return to the temperature at 20 °C, the CD spectra confirmed highly efficient reversible process. There are only small spectral differences observed, which could be assigned to the elevated presence of β-structure elements upon restoring initial conditions [2]. No spectral changes were observed for AMBN del E5 in the presence of Ca^2+^. The CD spectra temperature dependency of AMBN del E5 in 10 mM Ca^2+^ solution is almost the same as for AMBN del E5 in Ca^2+^-free solution, but the isosbestic point is red shifted to 210 nm (Figure S1). It can be concluded that AMBN del E5 behaves constantly as an IDP and this behavior is Ca^2+^ independent. [35].

To confirm the IDP character of all protein constructs (AMBN ISO I, AMBN ISO II, AMBN del E5) computational disorder prediction analysis by D2P2 (Database of Disordered Protein Predictions) was performed [36]. The results confirmed the IDP character of all studied proteins and did not reveal any significant differences between the AMBN ISO I and AMBN ISO II or AMBN del E5 (Figure S2, [36]).

### 2.2. AMBN ISO I and AMBN ISO II form Organized Oligomers

The measured autocorrelation functions G^(2)^ in Figure 1B for all studied molecules were obtained from a dynamic light scattering (DLS) experiment. The results show that the hydrodynamic radius of AMBN ISO I and AMBN ISO II in the Ca^2+^ buffer-free conditions is much larger than for AMBN del E5 construct and BSA used as a standard. This finding is fully consistent with observations by Wald et al. [2] who proved the importance of exon 5 for the oligomerization of AMBN ISO I. The analysis of autocorrelation functions (ACF) (Figure 1B) confirms the comparable behavior of both isoforms where AMBN ISO I is larger than AMBN ISO II. Unlike AMBN ISO I and AMBN ISO II, AMBN del E5 provided ACF corresponding to ACF of BSA standard used as a positive control to verify an AMBN del E5 monomeric character. The addition of Ca^2+^ into the solution caused only a small change of Rh of the both AMBN isoforms, keeping the size differences between AMBN ISO I and AMBN ISO II. All presented ACFs from DLS experiments were analyzed by second order cumulant fit and quantified by the corresponding z-averaged hydrodynamic radius [37] and polydispersity index (PDI) presented in Table 1.

### 2.3. AMBN Oligomers Partially Disintegrate by Increasing Temperature

AMBN ISO I and AMBN ISO II are able to form stable oligomers at room temperature. The interaction interface between AMBN monomers in the oligomer is maintained by exon 5 via non-covalent interactions of composing amino acids. Therefore, the question of the stability of these oligomers at temperature or aggregation properties can be answered by CD spectroscopy and DSF.

As follows from the CD spectra (Figure S1), there is a qualitative difference between the CD spectrum measured at 20 °C and 50 °C for both AMBN isoforms. Apparently, the higher content of β-elements indicating the tendency of aggregation appears at 50 °C and continuously rises up to 90 °C. Interestingly, all AMBN variants, including AMBN del E5, are to some extent fully reversible with temperature changes in the absence and presence of Ca^2+^.

Differential scanning fluorimetry (DSF) was used to illustrate changes in AMBN at the temperature range of about 20 °C and 50 °C. DSF data measured for AMBN ISO I, ISO II, and del E5 in the presence (10 mM CaCl_2_) and absence of Ca^2+^ were presented as the first derivative of the fluorescence emission ratio at 350 nm and 330 nm (F350/F330) (Figure 1C). It can be interpreted as disintegration peaks of the oligomeric forms of AMBN ISO I and AMBN ISO II with maxima around 35 °C. The disintegration occurs in both isoforms—AMBN ISO I and AMBN ISO II, and the presence of Ca^2+^ at 10 mM concentration does not significantly influences this behavior. In contrast, the spectrum of AMBN del E5 does not show a sign of any transition; its first derivative represents a noisy line, and T_m_ is not defined. This is in good agreement with the measured CD spectra of the AMBN del E5 showing an elevation of β-elements accompanying the aggregation. The data suggest that the AMBN ISO I and AMBN ISO II presented in oligomeric form first lose their oligomeric character with increasing temperature, and then, these non-oligomeric molecules begin to aggregate again, which most likely indicate a thermal non-specific aggregation.

**Figure 2:**
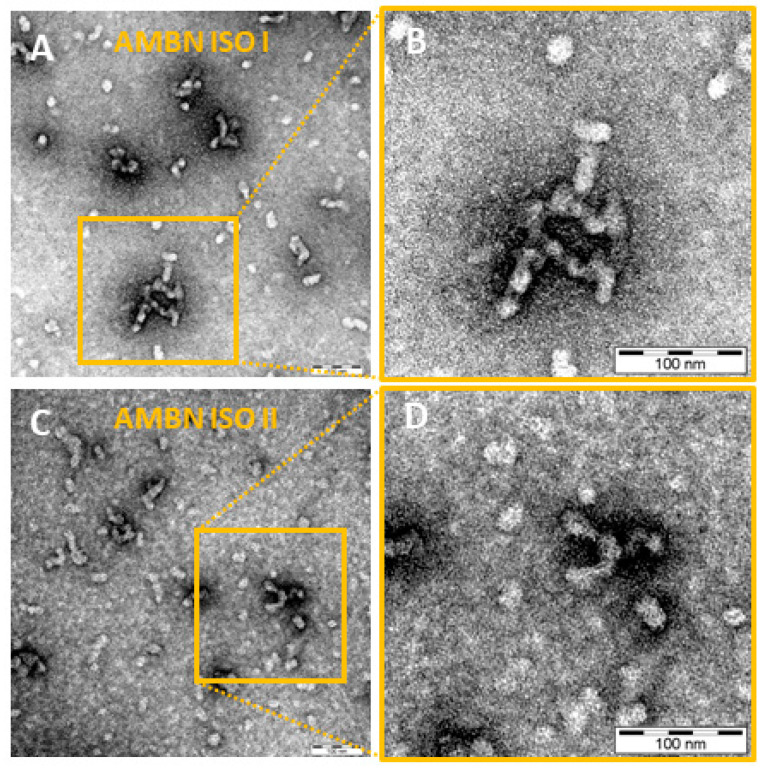
(**A**) Transmission electron micrographs of AMBN ISO I. Samples were examined at a magnification of 200,000×; scale bar = 100 nm. (**B**) Magnified detail of the AMBN ISO I presented in panel A. (**C**) Transmission electron micrographs of AMBN ISO II. Samples were examined at a magnification of 200,000×; scale bar = 100 nm. (**D**) Magnified detail of the AMBN ISO II presented in panel C.

**Figure 3:**
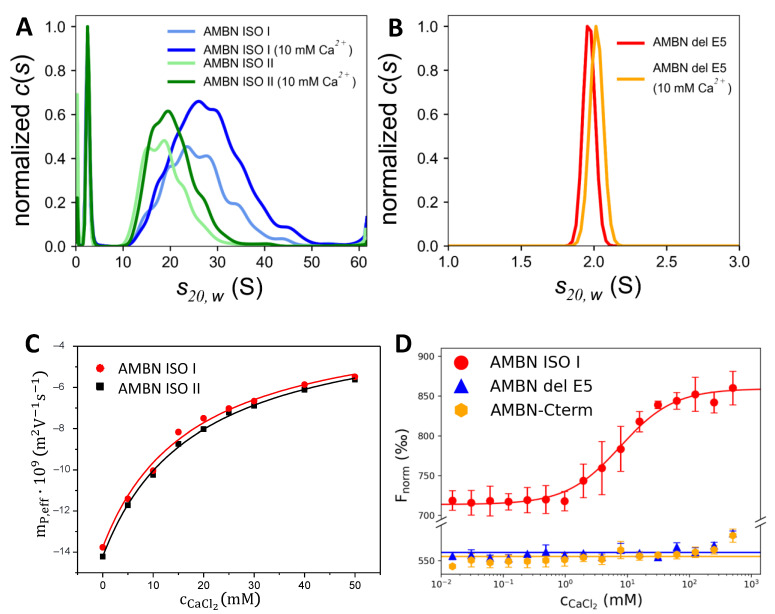
Sedimentation velocity analysis of (**A**) AMBN ISO I and AMBN ISO II; (**B**) AMBN del E5; in the absence or presence of 10 mM CaCl_2_. (**C**) Dependence of the effective electrophoretic mobility, *m_P,eff_*, of AMBN ISO I and AMBN ISO II proteins on the concentration of CaCl_2_ (c_CaCl_2__), in the background electrolyte (BGE) composed of 30 mM Tris, 25 mM acetic acid, pH = 7.4. (**D**) Microscale thermophoresis analysis of the interaction of the AMBN ISO I, AMBN del E5, and AMBN-Cterm with CaCl_2_. Titration of CaCl_2_ against a constant concentration of AMBN ISO I revealed a standard binding curve, while the titration of AMBN del E5 and AMBN C-term shows no binding.2.4. AMBN Oligomers of Both Isoforms Have a Heterogenous Character.

The transmission electron microscopy (TEM) micrographs in Figure 2 confirmed that both AMBN ISO I and AMBN ISO II form oligomers, which are heterogeneous in size. The observation is supported by results from analytical ultracentrifugation (AUC) studies (see Results in Section 2.4, Figure 3A) and corresponds to the results published by Wald et al. [2].

Sedimentation velocity analysis performed by the analytical ultracentrifugation (AUC) technique confirmed that the presence of exon 5 in AMBN ISO I and AMBN ISO II is a necessary element for the substantial oligomerization of both isoforms. While a broad range of oligomeric states can be observed for full-length AMBN isoforms (Figure 3A), a monodisperse distribution of the sedimentation coefficient (S) was observed for AMBN del E5. The molecular weight of AMBN del E5 predicted from this measurement was 42 kDa, corresponding well to the expected 41 kDa calculated from the protein amino acid sequence (Figure 3B). The fitted value of the frictional coefficients ratio f/f_0_, describing asymmetry of the sedimenting particle [38], was for AMBN del E5 determined as 2.2, clearly suggesting its intrinsically disordered character. Similar values were fitted for the monomeric species present in the samples of both AMBN isoforms (Table 2). Their oligomeric forms span a range of values from 10 to 40 S for AMBN ISO I (on average 26.1 or 28.5 S in absence or presence of Ca^2+^, respectively) and from 10 to 30 S for AMBN ISO II (on average 19.2 or 20.9 S in absence or presence of Ca^2+^, respectively). Thus, the average values correspond to an approximate molecular weight of 1.30 MDa or 1.54 MDa for AMBN ISO I (with an average oligomer size of 28 or 33 monomers) in the absence or presence of Ca^2+^, respectively, and 855 kDa or 900 kDa for AMBN ISO II (with an average oligomer size of 19 and 20 monomers) in the absence or presence of Ca^2+^, respectively. The f/f_0_ ratio of 1.6 to 1.7 fitted for the oligomeric species suggests that the oligomeric states might be slightly more compact due to the mutual interaction of the AMBN protein in both isoforms.

The AUC profile for AMBN ISO I and AMBN ISO II changes in the presence of Ca^2+^ as demonstrated by values for hydrodynamic properties of both monomeric (AMBN del E5) and oligomeric forms of AMBN. While for the AMBN del E5, only a small conformational change upon the addition of the Ca^2+^ was observed with its sedimentation coefficient shifting from 1.98 to 2.03 S, and the fitted f/f_0_ ratio decreasing from 2.2 to 2.1, a considerably bigger change was observed for both AMBN ISO I and AMBN ISO II oligomers. The oligomeric species in the presence of Ca^2+^ exhibited a higher level of compactness as evidenced by a small but consistent decrease of the fitted f/f_0_ ratios (for AMBN ISO I decrease from 1.70 to 1.64 and for AMBN ISO II decrease from 1.72 to 1.63) as well as their Stokes hydrodynamic radii (Table 2), thereby sedimenting faster with higher values of the observed sedimentation coefficient (Figure 3A).

### 2.4. AMBN Binds Ca^2+^ Only in Its Oligomeric Form

The records of capillary electrophoresis (CE) analyses of AMBN ISO I and AMBN ISO II proteins are presented in Figure S3. CE analyses in the buffered background electrolyte (BGE) containing Ca^2+^ showed that the migration times of proteins were increasing more significantly than the migration times of the electroosmotic flow marker dimethylsulphoxide (DMSO) with the increasing concentration of Ca^2+^ (Figure S3). This indicates an interaction between the proteins and Ca^2+^. The dependences of the effective electrophoretic mobilities of AMBN proteins on the concentration of Ca^2+^ in the BGE are shown in Figure 3C. The apparent binding constants K_b_ and their reciprocal values (apparent dissociation constants, K_D_) and ionic mobilities of the protein–Ca^2+^ complexes obtained by regression analysis of CE data are presented in Table 3. The CE records and data in Table 3 show that AMBN ISO I and AMBN ISO II proteins interact with Ca^2+^ with the apparent dissociation constant (K_D_) of the protein-Ca^2+^ (1:1) complexes similarly for both AMBN isoforms: AMBN ISO I (K_D_ = 17 mM) and AMBN ISO II (K_D_ = 18 mM). These relatively high values of K_D_ (low values of Kb, 54.4–56.8 L/mol, see Table 3) show that the interactions of AMBN ISO I and AMBN ISO II protein with Ca^2+^ cations are relatively weak. They are much weaker than the Ca^2+^ complexes with e.g., C-reactive protein, K_D_ = 59 µM [39]; on the other hand, there is a spectrum of secretory calcium-binding phosphoproteins (SCPPs) in wide range of affinities from nM to mM [40].

To confirm the above obtained values by CE, we used the microscale thermophoresis (MST) method to determine binding properties of at least one of the isoforms, AMBN ISO I, which represents the oligomeric form of AMBN. For comparison, we took the AMBN del E5 construct, representing the monomeric form of AMBN as the control. To complete the characterization of Ca^2+^-binding properties of AMBN, we analyzed AMBN-Cterm (exon10-exon13), which is highly negatively charged and does not form oligomers. An interaction between AMBN ISO I and Ca^2+^ was clearly confirmed, whereas for AMBN del E5–Ca^2+^ and AMBN-Cterm-Ca^2+^, no interaction was detected (Figure 3D). Therefore, we conclude that the oligomeric form of AMBN proteins is the major condition for Ca^2+^ binding. Although the dissociation constant (K_D_) for AMBN ISO I was determined in the low mM range (7.7 mM), it should be noted that the value is apparently the average of K_Ds_ due to the oligomerization heterogeneity of the AMBN ISO I. It is known that affinities for cellular proteins binding Ca^2+^ span the range from nM to mM [40,41,42,43], which is in agreement with our determined K_D_.

## 3. Discussion

The main determinant of the properties of AMBN ISO I and AMBN ISO II is their IDP character [1]. However, many AMBN-associated functions can be deduced from its behavior under physiological conditions in which the presence of Ca^2+^ [44] is frequent. The fact that AMBN is present in two isoforms—AMBN ISO I and AMBN ISO II—suggests that they could have different roles not only during the enamel formation but also during specific cellular functions. Different AMBN mRNA splicing resulting in the presence of AMBN ISO I and ISO II may lead to their various proteolytic profiles and interaction properties, which may lead to products with different functions [45,46]. We compared a large spectrum of AMBN ISO I and ISO II properties, which both have been shown to exist under physiological conditions in oligomeric states. This is in contrast to AMBN del E5 present only as a monomer. Still, AMBN ISO I and II as well as AMBN del E5 are preserving their IDP character in all performed CD experiments (without and in the presence of Ca^2+^ cations), as was confirmed by CD spectroscopy (Figure 1A). It is also in a good agreement with an analysis of computational disorder prediction by D2P2 (Figure S2).

The oligomeric and heterogeneous characters of AMBN ISO I and ISO II were further confirmed by DLS in the Ca^2+^ buffer-free conditions as well as in the presence of Ca^2+^. In addition, there appear to be small differences in the size and distribution of AMBN oligomers of both isoforms (AMBN ISO I creates larger oligomers than AMBN ISO II), as confirmed by TEM. It should be mentioned that we have to take into account the detection limit of the TEM resolution (Figure 2). This is in contrast to the behavior of AMBN del E5 confirmed by DLS and AUC methods, which exhibit monomeric protein properties (Figure 1B). AUC was employed to characterize the oligomeric state of AMBN and its variants. As clearly demonstrated by the presented results, the AMBN del E5 retained its monomeric character even in the presence of Ca^2+^, which is in sharp contrast to the observable effect of Ca^2+^ on AMBN ISO I and AMBN ISO II. We again assigned this difference to the oligomeric nature of both isoforms and confirmed that exon 5 is the most important prerequisite for oligomerization. AUC also provided results indicating the effect of Ca^2+^ demonstrated by the increased compactness of AMBN ISO I and ISO II. However, it is difficult to discern whether this behavior was due to a conformational change of the same oligomeric species or due to a shift in distribution toward higher AMBN oligomers caused by the Ca^2+^.

The temperature response of AMBN proteins was studied by CD spectrometry and DSF. DSF is commonly used to determine the melting temperature of denaturated protein [47]. In our case, we addressed a question of disintegration of the oligomeric state of AMBN. As follows from the temperature-dependent CD spectra, an interesting temperature interval was identified between 20 and 50 °C. The DSF results show similar behavior of both AMBN isoforms under two different conditions (Ca^2+^ free and 10 mM Ca^2+^ solutions) and suggest that there is a transition state attributable to the disintegration of AMBN oligomeric states into smaller oligomers and/or monomers. Both the CD as well as DSF spectra show the formation of higher AMBN aggregates upon increasing temperature, which was presumably formed non-specifically.

**Figure 4:**
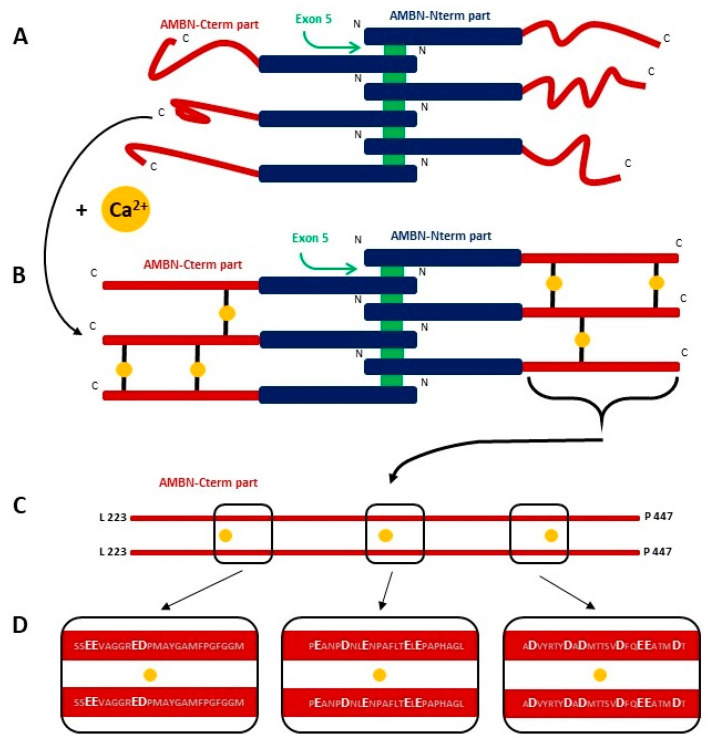
The schematic view of possible AMBN oligomerization state. AMBN monomers forming oligomers (e.g., hexamer). The blue rectangle illustrates AMBN-Nterm, while the green rectangle shows oligomerization inducing exon 5. Red lines show the possible arrangements of AMBN-Cterm in the absence of Ca^2+^ (**A**) and in the presence of Ca^2+^ (**B**) with yellow circles representing Ca^2+^. Black lines represent possible Ca^2+^ binding to AMBN-Cterm. (**C**) Schematic detail of possible Ca^2+^-binding sites of two C-termini of AMBN. Black rectangles represent sequences with a high abundance of negatively charged amino acids. (**D**) Detail view of potential Ca^2+^-binding sites at AMBN C-termini is shown in panel C. Amino acid sequences of AMBN-Cterm are in black rectangles. Negatively charged amino acids as a high preferable binding sites are bold white.

The final step of the study was to investigate the binding affinity of Ca^2+^ to AMBN isoforms. We performed CE and MST to determine the dissociation constants of Ca^2+^ complexes. From the CE results, we can conclude that both AMBN isoforms act as low affinity Ca^2+^-binding proteins with the similar K_D_. The MST experiment confirmed the binding of Ca^2+^ to AMBN ISO I, whereas no binding of Ca^2+^ was observed for AMBN del E5 and AMBN-Cterm, representing the monomeric states. It seems to be clear to conclude that the oligomeric state of AMBN is crucial for Ca^2+^ binding. Interestingly, the MST experiment determined a similar binding constant as determined by CE (for AMBN ISO I). To establish a schema of the potential mechanism of Ca^2+^ binding to AMBN oligomers, we propose a model of the oligomeric arrangement of AMBN in the absence and presence of Ca^2+^ (Figure 4). AMBN in solution without Ca^2+^ creates stable oligomers with flexible and dynamic AMBN-Cterm parts due to their disordered character. In the presence of Ca^2+^, the oligomeric form of AMBN becomes less dynamic at AMBN-Cterm due to stabilization introduced by interchain interactions of AMBN C-termini facilitated by Ca^2+^. This is in qualitative agreement with the AUC results, which show that both AMBN isoforms are slightly more compact in the presence of Ca^2+^ (Figure 2A).

The reason why we studied both isoforms and their spatial organization is that these extra 15 amino acids presented in Ambn ISO I lie just next to the exon 5 sequence, which is known to be the only element responsible for oligomerization. To use an analogy, the deletion of exon 5 (37 amino acids) completely removes the ability of the Ambn to create oligomers. The sequence of these 15 amino acids just next to the exon 5 terminus seems to be a good candidate for modulation of the oligomerization but to a lesser extent than exon 5 itself. Additionally, the sequence composition of these 15 amino acids is rich for tyrosines known for their role in protein–protein interactions. Whether the extension of exon 5 completely missing in ISO II could influence the oligomerization, size, or their tendency to aggregate was not clear, and we had to explore the possible consequences of the missing part of the sequence experimentally. We conclude that this is an extra element of modulation of oligomerization level.

## 4. Materials and Methods

### 4.1. Design of AMBN Plasmid Constructs

The expression vectors encoding AMBN ISO I, AMBN del E5, and AMBN-Cterm were kindly provided by the Institute of Microbiology of the CAS (Prague, Czech Republic) [2]; the vector encoding AMBN ISO II was obtained from the University of Chemistry and Technology (Prague, Czech Republic). The cDNA of AMBN were subcloned into the pET28b vector in fusion with 6xHis and the thioredoxin tags (Trx) removable with Tobacco Etch Virus (TEV) protease (for details, see [2]).

### 4.2. Protein Expression and Purification

AMBN ISO I (UniProtKB-Q9NP70-1), AMBN ISO II (UniProtKB-Q9NP70-2), AMBN del E5, and AMBN-Cterm were produced as recombinant proteins in Escherichia coli BL21(DE3) cells. Cultures were grown in Luria-Bertani (LB) broth with kanamycin (30 µg/mL) at 37 °C. The expression was induced by 0.5 mM isopropyl-β-D-thiogalactopyranoside and cultivation continued for 20 h at lower temperatures (14 °C for AMBN ISO I, 16 °C for AMBN ISO II, 30 °C for AMBN del E5, 15 °C for AMBN-Cterm). Cells were harvested by centrifugation, resuspended in a phosphate buffer: 50 mM Na_2_HPO_4_ (pH = 8.0), 50 mM NaCl, 0.1% 2-mercaptoethanol, and stored at −80 °C. Following thawing, the cells were sonicated at 4 °C, centrifuged for 1 h at 21,000× *g*; then, 8 M urea was added into the lysate. The lysate was purified by metal affinity chromatography using Chelating Sepharose Fast Flow resin (GE Healthcare Bio-Sciences AB, Uppsala, Sweden) charged with Ni^2+^ ions in an equilibration buffer (8 M urea, 50 mM Tris-HCl (pH = 7.4), 600 mM NaCl, and 20 mM imidazole). The bound proteins were eluted from the column by elution buffer containing 600 mM imidazole, and following renaturation, His-tagged TEV protease was added to the sample to remove His and Trx tags from AMBN proteins. Then, the whole reaction mixture was loaded again on the top of the HisTrap HP column (GE Healthcare Bio-Sciences AB, Uppsala, Sweden), and eluted AMBN ISO I and AMBN ISO II proteins were dialyzed overnight at 4 °C against a loading buffer containing 10 mM Tris-HCl (pH = 7.4), 100 mM NaCl. Then, 10 mM ethyleneglycol-bis(beta-aminoethylether)-N,N’-tetraacetic acid (EGTA) was added to the mixture, and following 4 h incubation, the sample was dialyzed overnight at 4 °C against a loading buffer. AMBN del E5 and AMBN-Cterm were incubated with 10 mM EGTA for 4 h and then loaded on a Superdex 200 Increase 10/300 GL column (GE Healthcare Bio-Sciences AB, Uppsala, Sweden) equilibrated with 10 mM Tris-HCl (pH = 7.4), 100 mM NaCl. The mass of all recombinant proteins was determined as follows: AMBN ISO I = 46.71 kDa; AMBN ISO II = 45.00 kDa; AMBN del E5 = 41.09 kDa; AMBN-Cterm = 25.36 kDa. The purity of the final samples was analyzed by SDS-PAGE (Figure S4), and AMBN protein sequences were verified by MS.

### 4.3. Circular Dichroism Spectroscopy

CD spectra were collected on a Jasco-1500 spectropolarimeter equipped with a Peltier thermostated holder PTC-517 (JASCO Inc., Easton, MD, USA) at room temperature (RT) and/or additionally at the temperature range 5–95 °C with an increment of 5 °C using the following experimental setup: (i) for RT from 190 to 300 nm, 0.1 mm cylindrical quartz cell, standard instrument sensitivity, 1 nm bandwidth, a scanning speed of 5 nm/min, a response time of 16 s, and two spectra accumulations; (ii) temperature dependence from 195 to 280 nm, a 0.2 mm rectangular quartz cell, standard instrument sensitivity, a 1 nm bandwidth, a scanning speed of 10 nm/min, a response time of 8 s, and one accumulation. The temperature reversibility was checked by measurement of the chilled solution back to 5 °C. After baseline subtraction, the final data were expressed as molar ellipticities ϴ (deg·cm^2^·dmol^−1^) per residue.

The AMBN samples have been prepared in the absence of Ca^2+^, and the same batch have been used for analyses in the presence of 10 mM CaCl_2_. Samples were measured in buffer (10 mM Tris-HCl (pH = 7.4) with 100 mM NaCl) and in concentration 23 µM.

### 4.4. Dynamic Light Scattering

DLS experiments were performed using an RiNA Laser Spectroscater 201 (RiNa, Berlin, Germany). All samples were measured at 18 °C, using an angle of 90°. Each sample was measured 60 times, and the acquisition time was 2 seconds. AMBN ISO I, AMBN ISO II, and AMBN del E5 purified in the absence of Ca^2+^ at a concentration 14 µM were used for measurements, as well as BSA as a standard. All samples were analyzed in 10 mM Tris-HCl (pH = 7.4) with a 100 mM NaCl buffer. Measurements of these samples were also performed in the presence of 10 mM CaCl_2_, which was added directly to these samples 30 minutes before the experiment. Protein solutions have been centrifuged before each measurement at 4 °C. The measured raw G^(2)^ autocorrelation functions (ACF) were recorded and thoroughly examined for artefacts. Measurements with a residual baseline or anomalous photocurrent count rate were discarded. Individual autocorrelograms were fitted by second-order cumulant analysis providing z-averaged size and polydispersity index (PDI). Hydrodynamic radii (Rh) [37] were estimated using the Stokes–Einstein relation. The reported values of Rh and PDI were calculated as the arithmetic average over 40–60 analyzed autocorrelation curves. Their uncertainty was estimated as the standard errors of the mean (SEM).

### 4.5. Analytical Ultracentrifugation

Sedimentation velocity measurements were performed on an analytical ultracentrifuge ProteomeLab XL-I (Beckman Coulter, Indianapolis, IN, USA) [48]. AMBN ISO I, AMBN ISO II, and AMBN del E5 samples at 23 µM concentration purified in the absence of Ca^2+^ were analyzed in a 10 mM Tris-HCl (pH = 7.4), 100 mM NaCl buffer. Alternatively, CaCl_2_ was added to 10 mM final concentration prior to the analysis to these samples using the same batch of protein. Sedimentation velocity experiments were conducted at 20 °C at 25,000 (AMBN ISO I and AMBN ISO II) or 48,000 (AMBN del E5) rpm using double sector cells and an An50-Ti rotor. In total, 250 absorbance scans were recorded at 280 nm in 4 min intervals. Buffer density, protein partial specific volumes, values of sedimentation coefficients corrected to standard conditions, and particle dimensions were estimated in Sednterp v1.10 [49]. Data were analyzed in Sedfit v16.36 [50] using a continuous sedimentation coefficient distribution c(s) with bimodal f/f_0_ model yielding a separate f/f_0_ values for monomeric and oligomeric AMBN species. Figures were prepared in GUSSI v1.4.6 [51].

### 4.6. Differential Scanning Fluorimetry

A Prometheus nanoDSF instrument (NanoTemper Technologies, Munich, Germany) was used to determine the temperature dependence of the intrinsic dual-UV fluorescence change in tryptophan and tyrosine residues. Capillaries were loaded with 10 μL of protein sample at 14 µM concentration and emission wavelengths λ = 330 and 350 nm were measured. The experiment was performed at a heating rate of 1.5 °C/minute, resulting in a data point density of 15 points/°C. To determine the melting point temperature T_m_, the ratio of the fluorescence intensities at both emission wavelengths (F350/F330) was used. Its first derivative displaying a peak at the point of maximal slope, which corresponds for globular proteins to the T_m_, was plotted. AMBN proteins (AMBN ISO I, AMBN ISO II and AMBN del E5) were analyzed in a 10 mM Tris-HCl (pH = 7.4), 100 mM NaCl buffer. CaCl_2_ was added to 10 mM final concentration prior to the analysis of AMBN proteins.

### 4.7. Transmission Electron Microscopy

AMBN ISO I and AMBN ISO II proteins at a concentration of 1.3 µM were visualized in 10 mM Tris-HCl (pH = 7.4), 100 mM NaCl buffer by negative stain transmission electron microscopy. Parlodion-carbon-coated grids were floated on the top of a 5 uL drop of the sample for 5 min. Then, the grids were transferred on the top of drop 2% uranyl acetate, stained for 2 × 15 s, and dried. Photomicrographs were taken with a JEOL JEM-1011 electron microscope (Peabody, MA, USA) operated at 80 kV.

### 4.8. Capillary Electrophoresis

CE analyses were performed in P/ACE MDQ System (Beckman Coulter, Fullerton, CA, USA) using the software P/ACE System MDQ, the Karat version (Beckman), and the Clarity data station (DataApex, Prague, Czech Republic) for data acquisition and evaluation [52]. The CE analyzer was equipped with a UV-vis photodiode array spectrophotometric detector (190–600 nm) set at 200 nm wavelength. CE analyses were carried out in the internally untreated fused silica capillary, total/effective (to the UV-detector) length 405/302 mm, inside/outside diameter 50/375 µm, with outer polyimide coating (Polymicro Technologies, Phoenix, AR, USA). The buffered background electrolyte (BGE) was composed of 30 mM Tris and 25 mM acetic acid (pH = 7.4). DMSO has been used as an electroneutral marker of electroosmotic flow. The new capillary was consecutively washed with 1 M NaOH, water, and the BGE with a pressure of 1 bar for 10 min each, and conditioned in the BGE at a separation voltage of 20 kV for 20 min. Between analytical runs, the capillary was rinsed with the BGE with a pressure of 1 bar for 2 min. The analyses were performed at a constant temperature of 25 °C inside the capillary using the constant input power of 0.4 W and cooling the outer capillary wall by liquid coolant to the temperature 21.8 °C. Separation voltage was in the range of 10.6–24.0 kV, and electric current varied between 17.1 and 37.9 µA. AMBN ISO I and AMBN ISO II were analyzed in a 10 mM Tris-HCl (pH = 7.4), 100 mM NaCl buffer. The concentration of AMBN proteins was 13 µM.

### 4.9. Microscale Thermophoresis

MST measurement and analysis of Ca^2+^-binding properties of AMBN ISO I, AMBN del E5, and AMBN-Cterm was performed as follows: a series of CaCl_2_ dilutions (500–0.015 mM) were incubated with AMBN ISO I (1.3 µM), AMBN del E5 (3.4 µM), and AMBN-Cterm (3.2 µM) in 10 mM Tris-HCl (pH = 7.4), 100 mM NaCl, and 0.05% Tween 20 for 30 min at 26 °C. The reactions were measured in Label-Free Premium capillaries on a Monolith NT. Label-Free instrument (NanoTemper Technologies, Munich, Germany) using 25% or 30% LED power and 40% MST IR laser power. All experiments were performed in triplicate. The plot was created using Matplotlib 3.2.1 [53]. The binding constants measured by MST were calculated using NTAnalysis software, v1.5.41 (Nanotemper Technology GmbH, Munich, Germany).

## 5. Conclusions

Human AMBN belongs to IDPs, which in fact significantly expands the portfolio of its functions with respect to the primary role of the protein involved in enamel formation. The presence of the two AMBN isoforms AMBN ISO I and AMBN ISO II has raised experimental questions as to whether biophysical properties are the main determinants of their different roles or whether there is another event to which the two isoforms respond differently. The results of this study only partially answered this question. Both isoforms exist only in the oligomeric form for which the exon 5 sequence is responsible. The isoforms bind Ca^2+^ with millimolar affinity, and it is only the oligomeric state that allows this binding. The deletion of exon 5 completely erases this ability, as demonstrated by MST experiments. The most important finding was that Ca^2+^ is able to bind to both isoforms, potentially suggesting that AMBN could act as a special Ca^2+^ buffer, especially in a Ca^2+^-abundant environment. We can conclude that AMBN binds Ca^2+^ only in its oligomeric form. This fact is demonstrated by the difference between AMBN ISO I, AMBN ISO II, and the apparently monomeric form of AMBN form lacking the exon 5 (AMBN del E5) and C-terminal part of AMBN (AMBN-Cterm). Both monomeric forms of AMBN (AMBN del E5 and AMBN-Cterm) do not bind to Ca^2+^.

Contrary to our expectations, Ca^2+^ does not appear to affect the formation of secondary or tertiary structures or their stabilization. The protein remains disordered, and Ca^2+^ binding is probably a transient process in which Ca^2+^ serve as proxies for intermolecular interactions between AMBN C-termini. This is supported by the fact that these Ca^2+^-mediated interactions lead to a more compact conformation of oligomeric AMBN species, as evidenced by their higher observed sedimentation coefficient values in the presence of Ca^2+^. With all the known properties of AMBN, it can be assumed that the only events that can be expected due to differences between both isoforms may be either their different proteolytic profiles presented by proteases (MMP20 and KLK4) or a different pattern of posttranslational modifications, particularly the phosphorylation [3]. Therefore, a detailed characterization of both processes is the aim of our further study.

## Figures and Tables

**Table 1:** Calculated hydrodynamic radii (Rh) [37] and polydispersity index (PDI) for AMBN ISO I, AMBN ISO II, AMBN del E5 variant of AMBN, and BSA in the absence or presence of Ca^2+^ ions. Numbers in parentheses represent the standard errors of the mean.

Protein	0 mM CaCl_2_		10 mM CaCl_2_	
	Rh (nm)	PDI	Rh (nm)	PDI
AMBN ISO I	24.0	0.13	21.6	0.09
	(0.2)	(0.02)	(0.2)	(0.08)
AMBN ISO II	17.7)	0.12	16.9	0.09
	(0.1)	(0.01)	(0.1)	(0.01)
AMBN del E5	5.7	0.06	5.7	0.06
	(0.05)	(0.01)	(0.05)	(0.01)
BSA	5.03	0.30	NA	NA
	(0.03)	(0.01)		

**Table 2:** Stokes hydrodynamic radii (R_S_) estimated from analytical ultracentrifugation (AUC) sedimentation velocity data for AMBN ISO I, ISO II, and del E5 variants and BSA in the absence or presence of Ca^2+^ ions.

Protein	0 mM CaCl_2_			10 mM CaCl_2_		
	R_S_ (nm)	s_20,w_ (S)	f/f_0_	R_S_ (nm)	s_20,w_ (S)	f/f_0_
AMBN ISO I monomer	4.44	2.53	2.09	4.30	2.61	2.02
AMBN ISO I oligomers	15.5	26.1	1.70	15.4	28.5	1.64
AMBN ISO II monomer	4.28	2.53	2.05	4.10	2.65	2.18
AMBN ISO II oligomers	13.5	19.2	1.72	13.0	20.9	1.63
AMBN del E5	4.98	1.98	2.20	4.85	2.03	2.10
BSA	3.38	4.60	1.27	NA	NA	NA

**Table 3:** The calculated apparent binding constants, K_b_, dissociation constant, K_D_, and the ionic mobilities, *m*_PM_, of the complexes of AMBN ISO I and AMBN ISO II proteins with Ca^2+^, and electrophoretic mobilities of these proteins in the background electrolyte free of Ca^2+^, *m*_p,eff,0_; R^2^, coefficient of determination.

Protein	K_b_ ± SD ^a^ (L/mol)	*m*_p,eff,0_ ± SD ^a^ (10^−9^ m^2^ V^−1^ s^−1^)	*m*_PM_ ± SD ^a^ (10^−9^ m^2^ V^−1^ s^−1^)	R^2^	K_D_ ± SD ^a^ (mol/L)
AMBN ISO I	56.8 ± 6.3	−13.8 ± 0.2	−2.39 ± 0.51	0.993	0.017 ± 0.002
AMBN ISO II	54.4 ± 3.0	−14.2 ± 0.2	−2.36 ± 0.27	0.998	0.018 ± 0.001

^a^ SD, standard deviation; *n* = 3.

## Supplementary Materials

The following are available online at https://www.mdpi.com/1422-0067/21/23/9293/s1.

## Abbreviations

Ambn	Ameloblastin protein
AMBN	Human ameloblastin protein
AMBN del E5	AMBN form lacking the exon 5
AMBN ISO I	Isoform I of AMBN
AMBN ISO II	Isoform II of AMBN
AMBN-Cterm	C-terminal part of AMBN
AMBN-Nterm	N-terminal part of AMBN
Amel	Amelogenin
AUC	Analytical ultracentrifugation
BSA	Bovine serum albumin
BGE	Buffered background electrolyte
Ca^2+^	Calcium ions
CD	Circular dichroism spectroscopy
CE	Capillary electrophoresis
D2P2	Database of Disordered Protein Predictions
DLS	Dynamic light scattering
DMSO	Dimethylsulphoxide
DSF	Differential scanning fluorimetry
EGTA	Ethyleneglycol-bis(beta-aminoethylether)-N,N’-tetraacetic acid
EMPs	Extracellular matrix proteins
Exon 5	Sequence encoded by exon 5 of AMBN
IDPs	Intrinsically disordered proteins
LB	Luria-Bertani broth
MMP20	Enamelysin
PDI	Polydispersity index
Rh	Hydrodynamic radii
RS	Stokes hydrodynamic radii
SCPPs	Secretory Calcium-Binding Phosphoproteins
SEM	Standard errors of the mean
TEM	Transmission electron microscopy
TEV	Tobacco Etch Virus
Trx	Thioredoxin tag
