# Supplementary material for: Characterization of AMBN I and II Isoforms and Study of Their Ca2+-Binding Properties
Source: Int J Mol Sci. 2020 Dec 5;21(23):9293. doi: 10.3390/ijms21239293 (PMC7730623; doi:10.3390/ijms21239293)
Supplement: Supplementary file 1 [file ijms-21-09293-s001.pdf]

SUPPLEMENTS

CD Spectroscopy

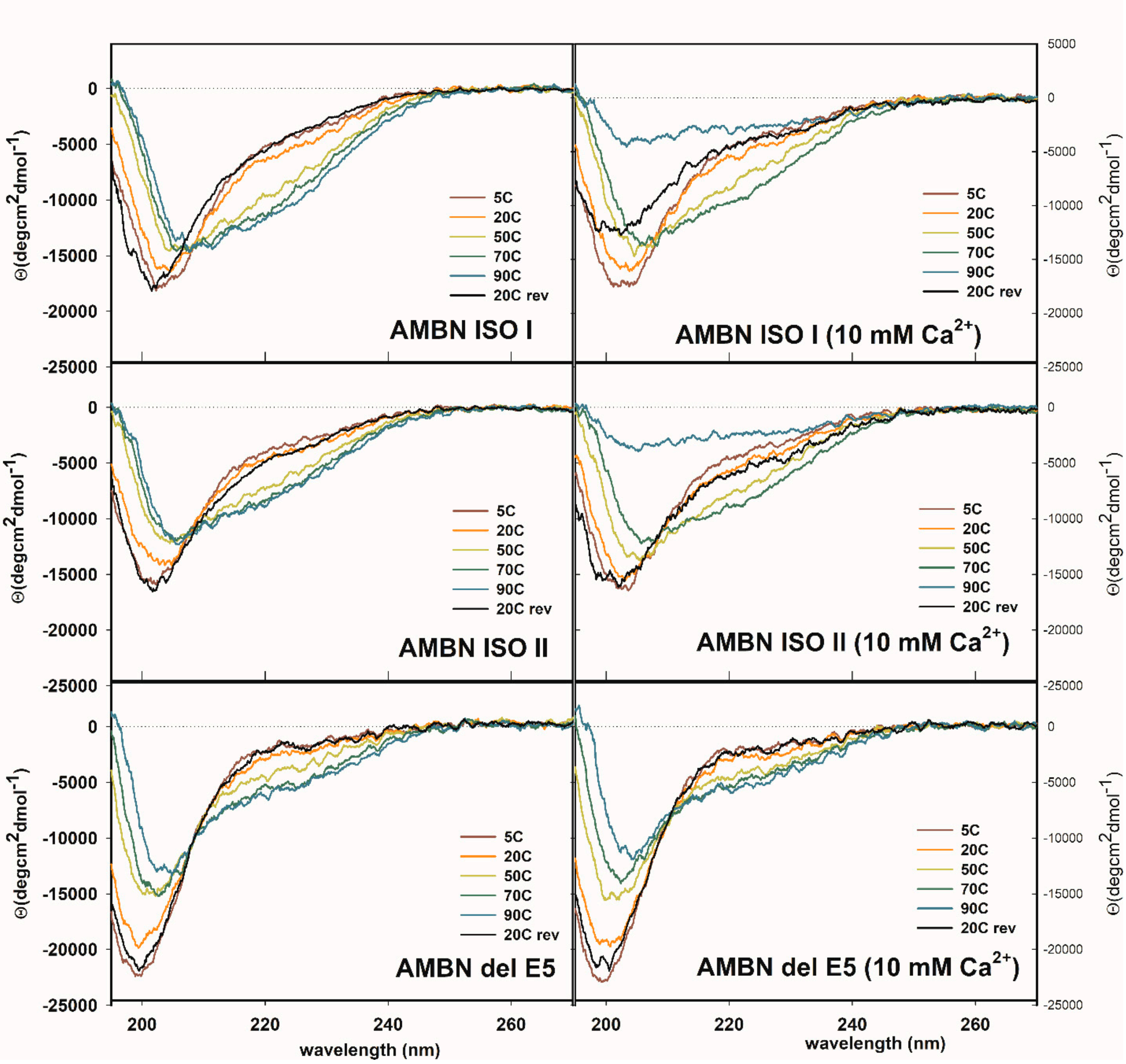

Figure S1. Temperature dependencies of CD spectra of AMBN ISO I, AMBN ISO II and AMBN del E5 without addition of Ca^{2+} ions and in presence of Ca^{2+} ions (10 mM $CaCl_2$).

Computational disorder prediction analysis

>sp|Q9NP70|AMBN_HUMAN Ameloblastin OS=Homo sapiens OX=9606 GN=AMBN PE=1 SV=1
MSASKIPLFKMKDLILILCLLEMSFAVPFFPQQSGTPGMASLSLETMRQLGSLQRLNTLSQYSRYGFGKSFNSLWMH
GLLPPHSSLPWMRPREHETQQYEYSLPVHPPPLPSQPSLKPQQPGLKPFLQSAAATTNQATALKEALQPPIHLGHLP
LQEGELPLVQQQVAPSDKPPKPELPGVDFADPQGPSLPGMDFPDPQGPSLPGLDFADPQGSTIFQIARLISHGPMPQ
NKQSPLYPGMLYVPFGANQLNAPARLGIMSSEEVAGGREDPMAYGAMFPGFGGMRPGFEGMPHNPAMGGDFTLEFDS
PVAATKGPENEEGGAQGSPMPEANPDNLENPAFLTELEPAPHAGLLALPKDDIPGLPRSPSGKMKGLPSVTPAAADP
LMTPELADVYRTYDADMTTSVDFQEEATMDTTMAPNSLQTSMPGNKAQEPEMMHDAWHFQEP

ENSP00000313809

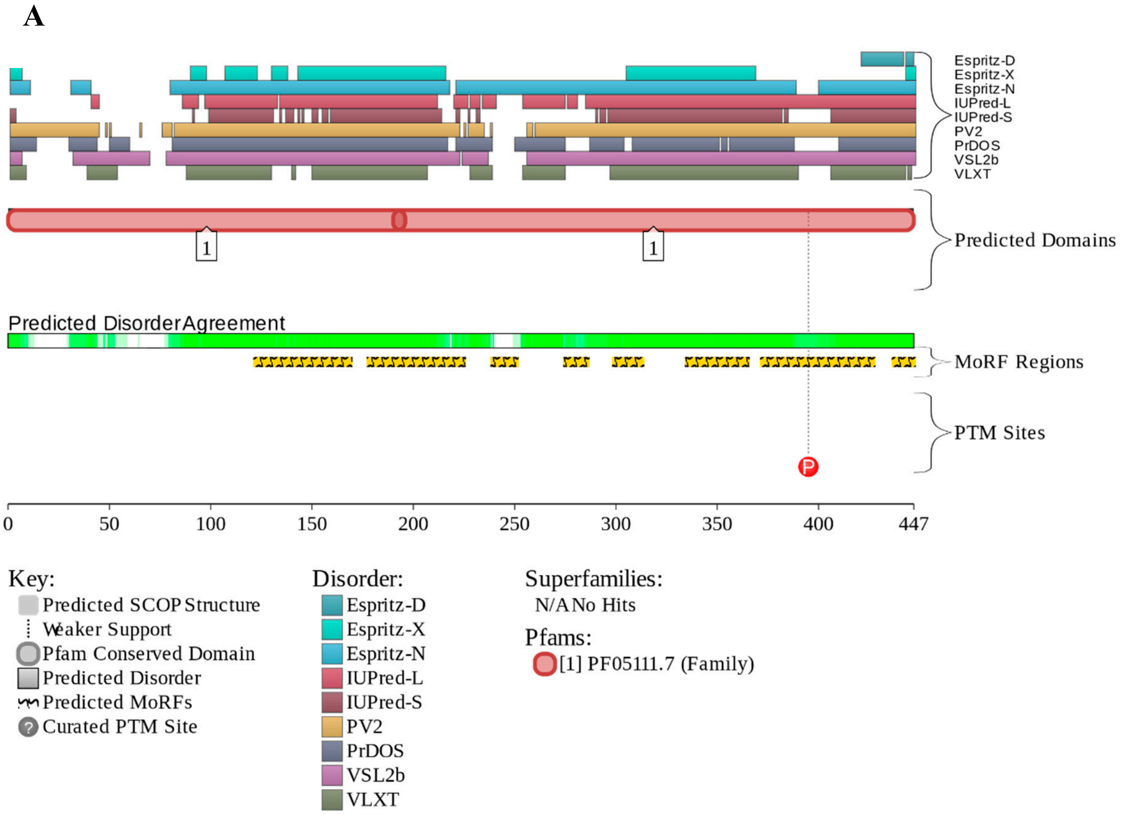

```
>sp|Q9NP70-2|AMBN_HUMAN Isoform 2 of Ameloblastin OS=Homo sapiens OX=9606
GN=AMBN
MSASKIPLFKMKDLILILCLLEMSFAVPFFPQQSGTPGMASLSLETMRQLGSLQRLNTLSQYSRYGFGKSFNSLWMH
GLLPPHSSLPWMRPREHETQQPSLKPQQPGLKPFLQSAAATTNQATALKEALQPPIHLGHLPLQEGELPLVQQQVAP
SDKPPKPELPGVDFADPQGPSLPGMDFPDPQGPSLPGLDFADPQGSTIFQIARLISHGPMPQNKQSPLYPGMLYVPF
GANQLNAPARLGIMSSEEVAGGREDPMAYGAMFPGFGGMRPGFEGMPHNPAMGGDFTLEFDSPVAATKGPENEEGGA
QGSPMPEANPDNLENPAFLTELEPAPHAGLLALPKDDIPGLPRSPSGKMKGLPSVTPAAADPLMTPELADVYRTYDA
DMTTSVDFQEEATMDTTMAPNSLQTSMPGNKAQEPEMMHDAWHFQEP
```

ENSP00000391234

B

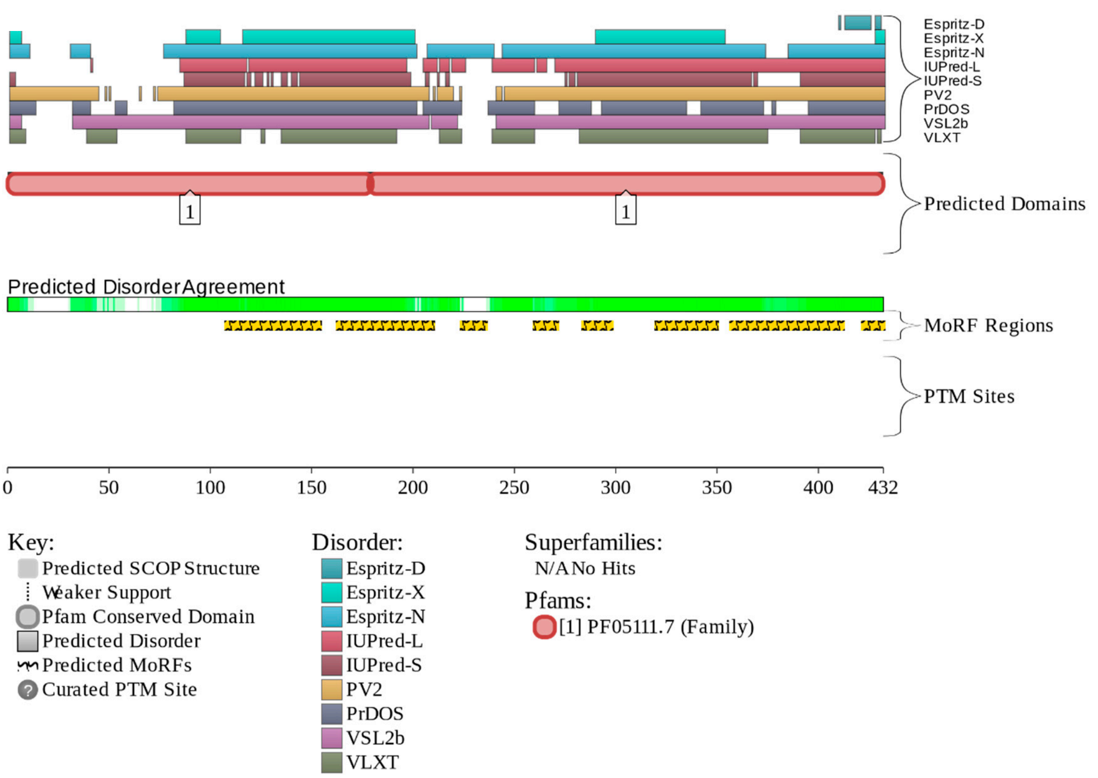

Figure S2. Computational disorder prediction analysis of AMBN ISO I **(A)** and AMBN ISO II **(B)**. Predictions have been analysed by D2P2: Database of Disordered Protein Predictions [37]. The scale of IDP character agreement is shown by light green colour.

CE experiments

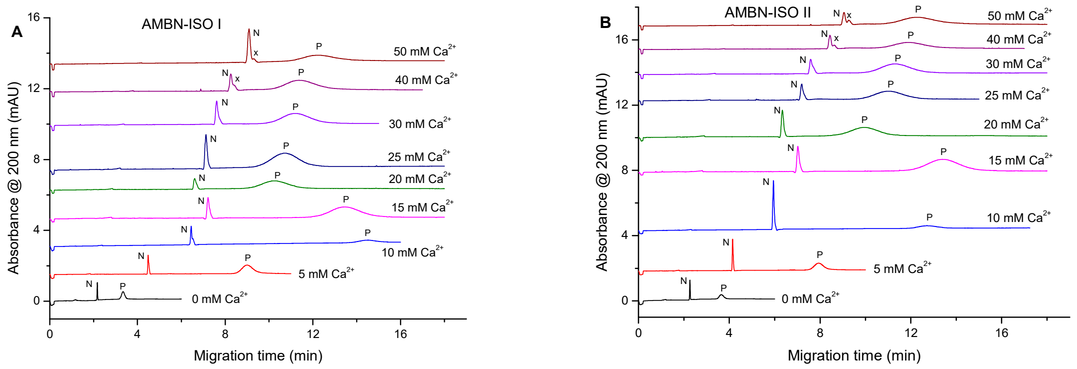

Figure S3. Records of CE analyses of AMBN ISO I **(A)** and AMBN ISO II **(B)** proteins in the background electrolyte (30 mM Tris, 25 mM AcOH, pH 7.4) containing variable concentration of calcium ions (0-50 mM). N, neutral marker of electroosmotic flow (DMSO); P, protein; x, non-identified component. In the CE analyses with Ca^{2+} ions concentration 15 mM and 20-50 mM, respectively, the electromigration velocity of all species was increased by the external pressure 138 Pa and 207 Pa, respectively, applied at the injection capillary end.

CE methods for determination of the apparent binding constant of protein complexes with calcium ions

Determination of the apparent binding constants of protein-metal ion complexes (taking into account concentrations instead of activities of the involved species) was based on measurement of the dependence of the effective electrophoretic mobility of protein P, $m_{\mathrm{P,eff}}$, on the concentration of the metal ion in the buffered background electrolyte (BGE). For the protein-metal ion complex with 1:1 stoichiometry ratio, this dependence was described by the equation [52]:

$$m_{P,\mathrm{eff}} = \frac{m_{P,\mathrm{eff,0}} + m_{PM} K_b c_M}{1 + K_b c_M} \qquad (1)$$

where $m_{P,\mathrm{eff,0}}$ is the effective mobility of a free (non-complexed protein (i.e. protein mobility in the BGE with zero concentration of the metal ion), m_{PM} is the ionic mobility of protein-metal ion complex PM, K_b is the apparent binding constant of the PM complex, and c_M is the equilibrium concentration of the metal ion in the BGE. With respect to the excess of metal ions in the BGE, the equilibrium concentration of metal ion is considered as approximately equal to the total metal ion concentration in the BGE.

The apparent binding constant, K_b, of the protein-metal ion complex was determined by plotting the effective mobilities of protein P against the concentration of the metal ion in the BGE and using non-linear regression analysis of the measured data according to equation (1).

The effective electrophoretic mobility of the protein P, $m_{P,\mathrm{eff}}$, was calculated from the CE experimental data using the equation (2)):

$$m_{P,eff} = \frac{L_{tot}L_{det}}{U_{sep}}\left(\frac{1}{t_{mig,P}} - \frac{1}{t_{eof}}\right) \qquad (2)$$

where L_{tot} and L_{det} are the total length of the capillary and the effective capillary length (from injection end to the detector), respectively, $t_{mig,P}$ is the migration time of the protein P, t_{eof} is the migration time of the electroneutral electroosmotic flow marker (dimethylsulphoxide (DMSO) in these measurements), and U_{sep} is the applied separation voltage.

SDS-PAGE analysis

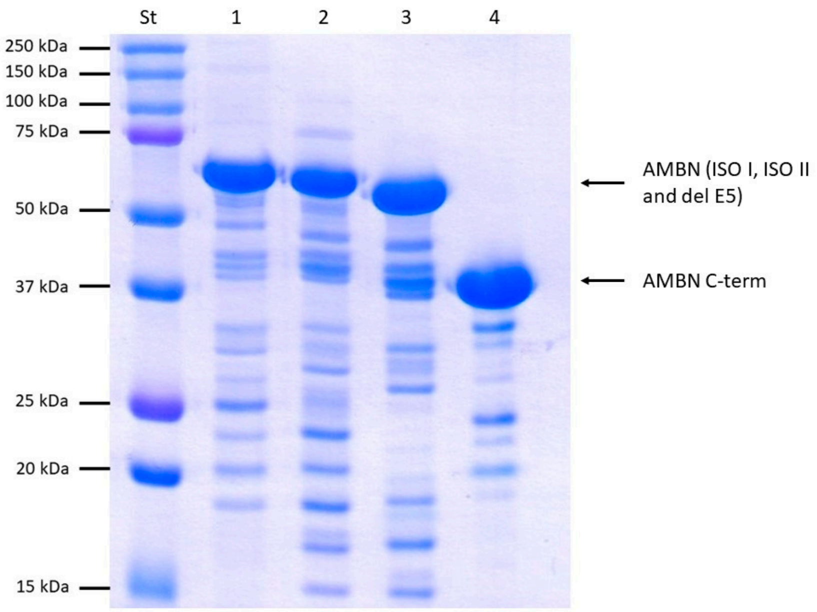

Figure S4. AMBN proteins analyses by SDS-PAGE using 12% polyacrylamide gel stained with Coomassie Blue. *Lane St*, molecular mass standards; *lane 1* AMBN ISO I; *lane 2*, AMBN ISO II; *lane 3*, AMBN del E5; *lane 4*, AMBN-Cterm. Calculated MW of AMBN proteins is lower than apparent size analyzed by SDS-PAGE. This phenomenon can occur at IDPs proteins. All AMBN proteins were analyzed by MS and their calculated MW was confirmed (data not shown).
